# Incidence, Risk Factors, Organism Types, and Outcomes of Catheter-Related Bloodstream Infections in Hemodialysis Patients

**DOI:** 10.7759/cureus.69554

**Published:** 2024-09-16

**Authors:** Adam Bitunguramye, Gerard Nkundimana, Ahmed M Aboubasha, Jules Kabahizi, William Rutikanga, Laetitia Nshimiyimana, Michel G Rafiki

**Affiliations:** 1 Epidemiology, University of Delaware, Newark, USA; 2 Internal Medicine and Nephrology, King Faisal Hospital, Kigali, RWA; 3 Nephrology, University of Rwanda, Kigali, RWA; 4 Statistics, King Faisal Hospital, Kigali, RWA

**Keywords:** risk factors, incidence, esrd, hemodialysis, catheter-related bloodstream infection

## Abstract

Background

Patients undergoing hemodialysis for End-Stage Renal Disease (ESRD) are at risk for Hemodialysis Catheter-Related Bloodstream Infections (CRBSIs). This study evaluates the incidence, risk factors, organism types, and outcomes of CRBSI in adult patients on maintenance hemodialysis at King Faisal Hospital, Rwanda.

Methods

This was a prospective cohort study of adult patients with end-stage renal disease undergoing hemodialysis via central venous hemodialysis catheters at King Faisal Hospital, Rwanda. Upon receiving the IRB approval, 81 eligible patients, women, and men aged between 19 and 74, were enrolled. Restricted Mean Survival Time (RMST) analysis evaluated the risk factors for CRBSI. The statistical significance was determined using p-values, with a cut-off of 0.05.

Results

The incidence of CRBSI was found to be 0.78 episodes per 1,000 catheter-days. Acute hemodialysis catheter type and anemia were associated with increased risk for CRBSI, with a P-value less than 0.05. In addition, all CRBSI cases were due to bacteria, with 52.63% gram-negative and 47.37% gram-positive. Out of 19 CRBSI events, nine cases (47.37%) required hospitalization with a median duration of seven days. Approximately half of the CRBSIs required catheter removal. No metastatic infection or death was noted.

Conclusion

The present study demonstrated that our hemodialysis unit has an incidence of 0.78 episodes per 1,000 catheter-days. Catheter type and anemia were significantly associated with CRBSI.

## Introduction

Catheter-related bloodstream infections (CRBSIs) are an essential cause of significant morbidity, mortality, and subsequent healthcare costs in end-stage renal disease (ESRD) patients undergoing maintenance hemodialysis [1-3]. Patients on hemodialysis endure infection rates that are more than 26 times higher than in the general population [2]. Infections in hemodialysis are the second leading cause of death and hospitalization [4]. Most CRBSIs are caused by skin flora colonizing the hemodialysis insertion site [1]. While it is well documented that arteriovenous fistula remains the preferred vascular access associated with better survival, quality of life, and low hospitalization rate, most end-stage renal disease patients continue to initiate dialysis using catheters [5-6].

The United States Renal Data System 2023 annual data report highlighted that in 2021, more than 85% of patients initiated hemodialysis with catheters, and nearly three-quarters of individuals did not have permanent access at the time of dialysis consideration [7]. In East Africa, hemodialysis remains the mainstay of renal replacement therapy, while the treatment of choice for end-stage renal disease, known as kidney transplantation, is a limited option. Advanced chronic kidney care is virtually unavailable, and most patients arrive in the hospital requiring urgent dialysis, which is started on catheters by a few trained healthcare professionals [8].

In a large cohort of 472 incident hemodialysis patients receiving first-ever tunneled hemodialysis catheters in the United States, catheters were plagued by high rates of infections. The median time to catheter-related bloodstream infection was 163 days, with 35% of patients infected at three months, 54% at six months, 79% at 12 months, and subsequent catheter failure in 45% [9]. Traditionally, gram-positive organisms, mainly *Staphylococcus aureus* and coagulase-negative *Staphylococci* are reported to account for the majority of CRBSIs in most studies, from 35 to 83.89% of isolates [10-11]. However, a retrospective cross-sectional study in Ethiopia recorded an increasing trend towards gram-negative predominance of up to 57% of cases [12]. Similarly, comparable findings are documented in Uganda, with gram-negative causative organisms accounting for 60.3 % of identified cases [13]. In the catheter-dependent hemodialysis population, CRBSIs can result in metastatic complications such as infective endocarditis, osteomyelitis, septic arthritis, septic shock, and epidural abscess from 5 up to 10% [10].

The most common risk factors for catheter-related bloodstream infection in patients undergoing hemodialysis are prolonged use of the dialysis catheter, prior history of CRBSIs, difficult insertion, methicillin-resistant *Staphylococcus aureus *(MRSA) carriage, and bacteremia three months before catheter insertion, diabetes mellitus, immunosuppression, hypoalbuminemia, iron overload, and recent surgery [14-17]. Furthermore, data on chronic kidney disease (CKD) are grossly unreliable in most sub-Saharan African countries because of a lack of renal registries; it is estimated that by the end of 2030, more than 70% of patients with ESRD will be in countries with similar backgrounds to those of Sub-Saharan Africa, where over 400 million people live on less than a dollar per day and have a gross domestic product (GDP) per capita of less than 1500$ per year [18-19]. In Rwanda, CKD prevalence accounts for 20%, and end-stage renal disease is the leading cause of hemodialysis, predominantly affecting young individuals aged between 35 and 57 years, with only 3% benefiting from kidney transplants [20-21].

Little is known about the epidemiology and risk factors of CRBSIs in Rwanda outside existing literature. Most end-stage renal disease patients initiate and maintain hemodialysis using hemodialysis catheters. Therefore, an in-depth understanding of the incidence, etiologies, and associated risk factors of CRBSIs could facilitate infection prevention and control (IPC) and develop best practice guidelines. This study investigated the incidence, risk factors, organism types, and outcomes of CRBSIs in adult patients undergoing maintenance hemodialysis at King Faisal Hospital, Rwanda, over 12 months between June 2023 and June 2024.

## Materials and methods

Study design and setting

A hospital-based, analytical, single-center prospective cohort study was conducted to determine the incidence, risk factors, types of organisms, and outcomes of catheter-related bloodstream infections in adult end-stage renal disease patients aged 18 years and older on maintenance hemodialysis using both tunneled and acute hemodialysis catheters. Study participants were monitored for CRBSIs from recruitment until the end of the study. End-stage renal disease patients were selected according to the definition of the Kidney Disease: Improving Global Outcomes (KDIGO) organization clinical practice guidelines for evaluating and managing CKD [22-23]. This study was conducted at King Faisal Hospital, Rwanda, a 160-bed multispecialty quaternary hospital located in Kigali, with a mandate to provide specialized healthcare in East and Central Africa. The hospital has an in-center hemodialysis unit located away from the inpatient building. It is equipped with 14 operational dialysis machines and staffed by 12 nurses, one medical officer, one nephrology fellow, and two nephrologists.

Study population

The study was conducted between June 1, 2023, and June 30, 2024, and included patients with permanent hemodialysis catheters inserted from January 1, 2019, onward but without any prior evidence of CRBSI.

Inclusion criteria

All end-stage renal disease patients aged 18 years and above on maintenance hemodialysis through central venous tunneled or acute hemodialysis catheters at King Faisal Hospital, Rwanda, during the study period were included.

Exclusion criteria

This study excluded adult end-stage renal disease patients who had CRBSIs before enrollment, those who lacked consent, individuals with an active arteriovenous fistula or arteriovenous graft, individuals with acute kidney injury, those with indwelling Foley catheters and coexisting intravascular devices, those with an identified alternative source of infection, as well as individuals under 18 years of age due to the poor representation of the pediatric population on chronic hemodialysis.

Data collection

Patients’ data were collected from the hospital management information system, medical records, and through direct patient inquiries using a prepared questionnaire for demographic information.

The dependent variable was the presence of catheter-related bloodstream infections. The independent variables included age, gender, anemia status, diabetes mellitus, hypoalbuminemia, hemodialysis catheter type, catheter insertion site, and the number of hemodialysis sessions per week. Anemia was defined as a hemoglobin (Hb) concentration of less than < 13g/dL for adult men and postmenopausal women and Hb under 12 g/dL in premenopausal women. Hypoalbuminemia was defined as a serum albumin of less than 3.5g/dL.

Additionally, microbiological information, catheter removal, loss of vascular access, length of hospital stays, intensive care unit admission, metastatic complications such as infective endocarditis, septic arthritis, vertebral osteomyelitis, and deaths were assessed to determine patients' clinical outcomes.

Definition of catheter-related bloodstream infection

In patients on hemodialysis with clinical manifestations of CRBSI, the diagnosis required concurrent blood cultures positive for the same organism from two different sites, obtained before antimicrobial therapy and without evidence of an alternative source of infection. This study considered paired blood cultures, with one sample obtained from a hemodialysis catheter hub and the other from either a peripheral vein or the hemodialysis circuit bloodline while the patient was on dialysis [24].

Primary and secondary objectives

The primary objective was to determine the incidence, risk factors, and organism types of catheter-related bloodstream infections in adults with End-Stage Renal Disease on maintenance hemodialysis. 

The secondary objective was to assess the clinical outcomes of catheter-related bloodstream infections.

Sample size

**Figure 1:**
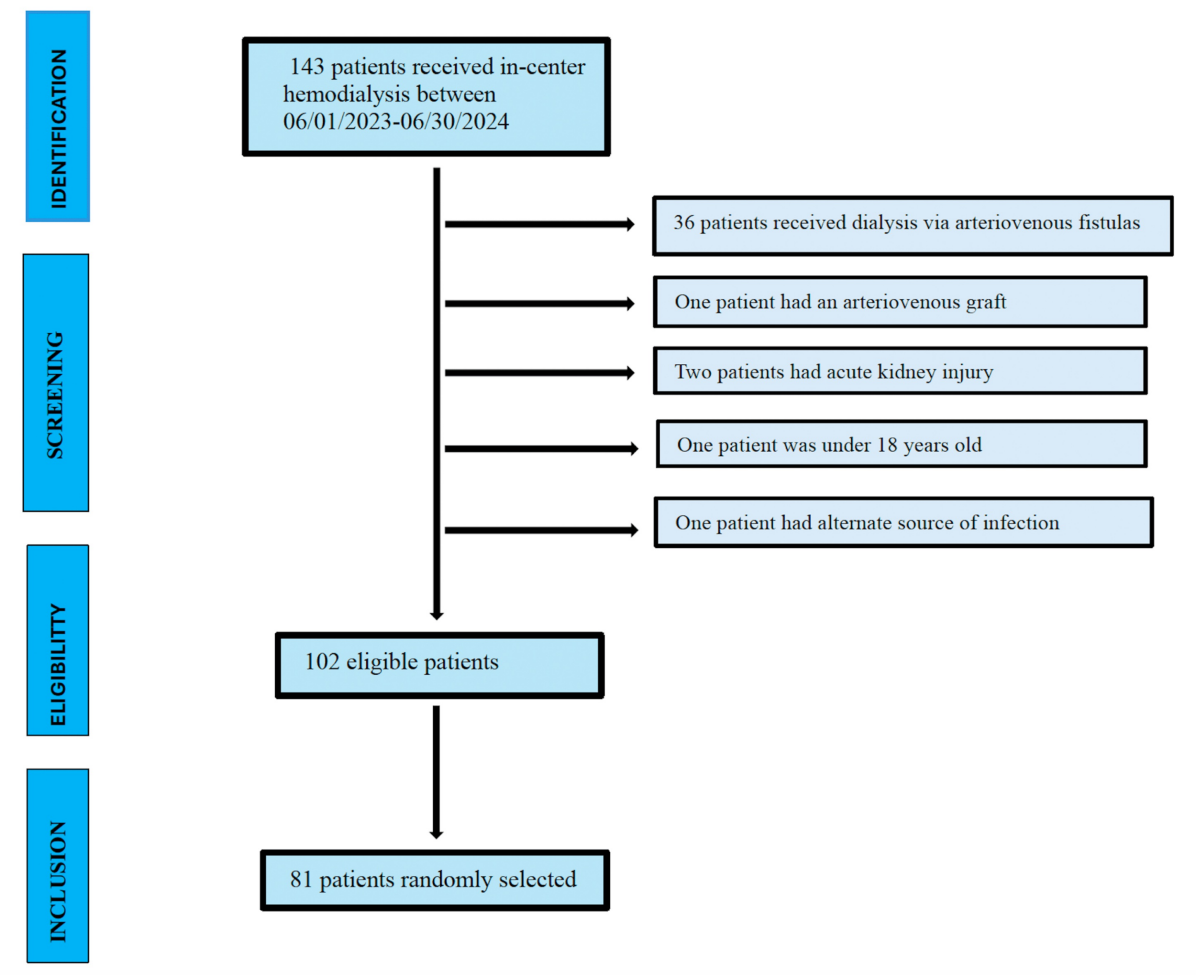
Schematic Representation of Patient Selection

Data were collected from all patients who met the study inclusion criteria. Cochran equation for finite population was used to determine our sample size. The sample size was adjusted to 81 study participants, assuming a confidence interval of 95% and ±5% precision, a population proportion variation of 50%. 143 patients received in-center -hemodialysis between June 1st,2023 and June 30th, 2024. Out of these, 41 patients were excluded from the study for the following reasons: 36 patients received dialysis via arteriovenous fistulas, one via arteriovenous graft, two had acute kidney injury, one had an alternate source of infection, and one was under 18 years old. Ultimately, 81 participants from the 102 eligible patients were selected for inclusion in the final study (Figure 1).

Bias and confounding approach

Eligible patients were randomly selected during data collection to reduce selection bias. Vital explanatory variables for catheter-related bloodstream infections (CRBSIs) were also stratified. Age and gender were accounted for in the fully adjusted model to control for potential confounding effects. Additionally, participants were grouped based on the presence of a baseline variable of interest, such as anemia, before hemodialysis catheter insertion to prevent misclassification (information bias). This ensured that any status changes would be noted during follow-up; however, no changes in diagnosis were observed, as most of these conditions are chronic.

Statistical analysis

Study data were collected and managed using Redcap. After cleaning, the final dataset contained no missing values, logical inconsistencies, or wild codes. For the operationalization of variables, only the variables of interest were retained, including hemodialysis catheter site and type, anemia, hypoalbuminemia, comorbidities, diabetes, number of hemodialysis sessions per week, age, and gender. The dates of hemodialysis catheter insertion, CRBSI event, and participant completion were primarily used to calculate the duration and incidence rate in the study. Still, they were not included in the model assessing the risk factors for CRBSI. Comorbidities included cancer, coronary heart disease, heart failure, chronic hepatitis B and C, dilated cardiomyopathy, and HIV. Age was kept a continuous variable, while most predictors were dichotomized into binary variables. There were no participants with iron overload, and only one patient had recent surgery; therefore, these two variables were not included in the model.

Most explanatory variables violated the proportional hazards assumption, making the Cox PH model unsuitable. The dataset was analyzed using Restricted Mean Survival Time (RMST) to assess the average time to developing CRBSIs across strata at τ = 180 days, meaning within the first six months of study enrollment. This cut-off was chosen because the median catheter duration for censored patients and those with CRBSIs were 191.5 days and 148 days, respectively. Additionally, a large prospective study conducted in the U.S. by Shingarev et al. [9], which included 472 patients receiving their first-ever tunneled catheters for hemodialysis initiation, found a median time of 163 days to the first CRBSI event, highlighting the clinical relevance of this period. All statistical analyses were conducted using SAS9.4/R4.3.2 software (SAS Institute, Cary, USA), with statistically significant differences at a p < 0.05.

Descriptive statistics were performed to analyze the CRBSI outcomes. The incidence density was calculated by dividing the number of incidents of CRBSI cases (19) by the total catheter days contributed by all participants (24,219 days). A Mann-Whitney U test was performed to compare the difference in length of hospital stay between patients with acute hemodialysis catheters and those with permanent hemodialysis catheters, as the assumptions for a parametric t-test were violated. The Shapiro-Wilk test indicated that the normality assumption was violated for the permanent hemodialysis catheter (p=0.00073), while Levene's test confirmed that the variances between the groups were not significantly different (p=0.7749).

Ethical consideration

Ethical clearance was obtained from the University of Rwanda, College of Medicine and Health Sciences (CMHS), and King Faisal Hospital, Rwanda Institutional Review Boards. The anonymity and privacy of study participants were preserved during study data collection and analysis.

## Results

During the study period, 81 eligible patients, both women and men aged between 19 and 74, were recruited between June 1, 2023, and June 30, 2024. Nearly all patients had health insurance coverage (99%). The median age of the study participants was 58 years, and the median time to the first episode of CRBSI was 148 days. The overall mean age was 52.85 years, with a standard deviation of 13.97 years (Table 1).

**Table 1:** Baseline Characteristics of the Study Population by CRBSIs Outcome (n=81) ***Other sites include Transhepatic and Translumbar. *Age represents the numerical age variable with means and standard deviation. ^&^Sessions per week is the number of hemodialysis sessions per week. CRBSI: catheter-related blood stream infection

	Overall cohort (n= 81)		CRBSIs(n=19)		No CRBSIs(n=62)		
	N	%	N	%	N	%	P-value
Catheter Type							
Acute hemodialysis catheter	26	32.10	7	36.84	19	30.65	0.6127
Permanent hemodialysis catheter	55	67.90	12	63.16	43	69.35	
Anemia							
No	9	11.11	0	0.00	9	14.52	0.0782
Yes	72	88.89	19	100	53	85.48	
Comorbidities							
No	65	80.25	17	89.47	48	77.42	0.2482
Yes	16	19.75	2	10.53	14	22.58	
Sessions per week^&^							
Two sessions	14	17.28	3	15.79	11	17.74	0.8439
Three sessions	67	82.72	16	84.21	51	82.26	
Gender							
Female	29	35.80	7	36.84	22	35.48	0.9140
Male	52	64.20	12	63.16	40	64.52	
Age*	52.85	13.97	50.16	12.16	53.68	14.47	
Diabetes							
No	47	58.02	11	57.89	36	58.06	0.9895
Yes	34	41.98	8	42.11	26	41.94	
Catheter Site							
Internal Jugular Vein	79	97.53	18	94.74	61	98.39	0.3697
Other sites ***	2	2.47	1	5.26	1	1.61	
Hypoalbuminemia							
No	75	92.59	17	89.47	58	93.55	0.5530
Yes	6	7.41	2	10.53	4	6.45	

The incidence rate of CRBSI was 0.78 episodes per 1,000 catheter-days. Over the first six months (tau = 180 days), patients with acute hemodialysis catheters developed CRBSI, on average, approximately 31 days earlier compared to those with permanent hemodialysis catheters. This finding was consistent in both the unadjusted (mean difference = -31.20 days, 95% CI: -56.00 to -6.40, P < 0.0001) and fully adjusted models (mean difference = -31.01 days, 95% CI: -54.27 to 7.75, P = 0.0090). Patients without anemia took approximately 19 days longer to develop CRBSIs in the fully adjusted model (mean difference = 18.99 days, 95% CI: 3.49 to 34.49, P = 0.0163), suggesting that those with anemia were more prone to develop CRBSIs approximately three weeks earlier, on average (Table 2).

**Table 2:** Restricted Mean Survival Time (RMST) Estimates for Risk Factors for Hemodialysis Catheter-Related Bloodstream Infections (CRBSIs) at τ= 180 days (n=81) Table 2 displays the results of statistical analyses evaluating the association between various risk factors (such as anemia, catheter type, hypoalbuminemia, and frequency of hemodialysis sessions) and the occurrence of catheter-related bloodstream infections (CRBSIs) among patients undergoing hemodialysis at King Faisal Hospital, Rwanda. The table includes adjusted and unadjusted restricted mean survival time (days), confidence intervals, and p-values for each risk factor. ^&^Non-internal jugular vein contains both transhepatic and translumbar sites. ^&&^Comorbidities include cancer, coronary heart disease, dilated cardiomyopathy, heart failure, hepatitis C Hepatitis B, and HIV. ^&&&^sessions represent the number hemodialysis sessions per week.  95% CI stands for 95% confidence interval.

	Unadjusted			Fully Adjusted		
	Estimate(days)	95% CI	P-value	Estimate(days)	95 % CI	P-value
Catheter type						
Acute hemodialysis catheter	-31.20	(- 56.00, -6.40)	< 0.0001	-31.01	( -54.27, -7.75)	0.0090
Permanent hemodialysis catheter	Ref	Ref		Ref	Ref	
Catheter site						
Internal jugular vein	9.18	(-20.26, 38.64)	0.5410	17.74	(-33.26, 68.75)	0.4954
Non-internal jugular vein^&^	Ref	Ref		Ref	Ref	
Hypoalbuminemia						
No	-14.04	(-24.39, -3.69)	0.0079	-11.17	(-28.79, 6.45)	0.2141
Yes	Ref	Ref		Ref	Ref	
Anemia						
No	15.18	(4.59, 25.77)	0.0050	18.99	(3.49, 34.49)	0.0163
Yes	Ref	Ref		Ref	Ref	
Diabetes						
No	-12.31	(-29.08, 4.46)	0.1502	-3.75	(-22.21, 14.70)	0.6901
Yes	Ref	Ref		Ref	Ref	
Comorbidities^&&^						
No	3.09	(-22.13, 28.32)	0.8100	5.97	(-18.96, 30.90)	0.6389
Yes	Ref	Ref		Ref	Ref	
Sessions^&&&^						
Two sessions	6.39	(1.45, 26.51)	0.0287	9.34	(-3.75, 22.43)	0.1619
Three sessions	Ref	Ref		Ref	Ref	
Sex						
Female				-6.64	(-27.69, 14.41)	0.5363
Male				Ref	Ref	
Age				0.47	(-0.24, 1.17)	0.1932

Additionally, patients without hypoalbuminemia developed CRBSI on average 14 days earlier in the unadjusted model (mean difference = -14.04 days, 95% CI: -24.39 to -3.69, P = 0.0079), but this was not significant in the fully adjusted model (mean difference = -11.17 days, 95% CI: -28.79 to 6.45, P = 0.2141). Moreover, those undergoing two dialysis sessions per week had a non-significant delay in developing CRBSI compared to those with three sessions, with the unadjusted model showing a mean difference of 6.39 days (95% CI: 1.45 to 26.51, P = 0.0287), while the fully adjusted model showed a mean difference of 9.34 days (95% CI: -3.75 to 22.43, P = 0.1619), which was not statistically significant. Therefore, the variables for dialysis sessions and hypoalbuminemia were not statistically significant in the fully adjusted model, likely because other covariates may have explained the variation in time to develop CRBSI (Table 2).

**Figure 2:**
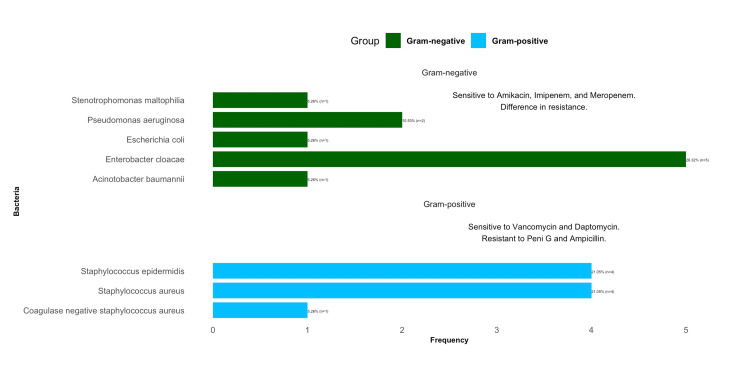
Bacterial Frequencies: A Synopsis of Antibiotic Sensitivity Analysis Among Patients with Hemodialysis Catheter-Related Bloodstream Infections (n=19)

In addition, only bacterial isolates were identified. Gram-negative organisms were responsible for most CRBSIs, accounting for 52.63% of cases, while gram-positive organisms were implicated in 47.37% of all isolates. *Enterobacter cloacae* was the predominant bacterium, representing 26.32% of all cases. CRBSIs due to gram-positive organisms were caused by methicillin-susceptible *Staphylococcus aureus* (21.05%), coagulase-negative *Staphylococcus aureus* (5.26%), and *Staphylococcus epidermidis* (21%), as shown in Figure 2.

Among patients with CRBSI, 47.37% required hospitalization with a median hospital stay of seven days, 10.53% had Intensive Care Unit (ICU) admission, and 47.37% had their hemodialysis catheter removed, mainly due to CRBSI from *Pseudomonas aeruginosa* and *Staphylococcus aureus* infections. There were no cases of loss of vascular access, metastatic complications, or deaths attributed to CRBSI.

The mean length of hospital stay in CRBSI patients with acute hemodialysis catheters was 4.86 days, while that of patients with permanent hemodialysis catheters was 3.5 days. Given the violation of normality and the small sample size (n = 19), the non-parametric Mann-Whitney U test was chosen as a more appropriate method. The test showed no statistically significant difference between the two groups (W = 50, p = 0.492), using a continuity correction due to ties.

## Discussion

The present study showed 19 CRBSIs, resulting in an incidence density of 0.78 cases per 1,000 catheter-days. The Restricted Mean Survival Times (RMST) analysis demonstrated a statistically significant association between anemia, catheter type, and CRBSI (p-value < 0.05) in both the unadjusted and fully adjusted models. Gram-negative organisms were responsible for most CRBSI, accounting for 52.63%, and gram-positive implicated in 47.37% of all cases. Among patients with CRBSI, 47.37% required hospitalization with a median hospital stay of seven days, and 47.37% required definitive catheter management by removal. 

This present study demonstrated that the hemodialysis unit achieved a low baseline incidence compared to the AZEPTIC trial conducted by Maki et al. in the United States in 25 outpatient hemodialysis units using methylene lock solution, which demonstrated that review, retraining, and implementation of best central venous catheters care practices can achieve a low baseline of 0.78 events per 1,000 catheter-days; this recommendation was further endorsed by updated National Kidney Foundation Kidney Disease Outcomes Quality Initiative (NKF KDOQI) clinical practice guideline for vascular access updated in 2019 [24-25]. However, the incidence in this study is twice as high as that of an incidence density of 0.40 episodes per 1,000 catheter-days, as described by Almenara-Tejederas et al. Their 14-year retrospective observational cohort study was conducted in Spain between 2005 and 2019 [26].

Tal et al. described a comparable incidence of 0.76 episodes per 1,000 catheter-days In a retrospective study of 60 patients in the Dominican Republic carried out between 2016 and 2019 using non-sided-hole tunneled hemodialysis catheters [27]. In contrast, higher incidences of CRBSIs are reported in Uganda and Ethiopia dialysis units, with 5.2 and 7.74 cases per 1,000 catheter-days, respectively [12-13].

A meta-analysis study conducted in 2023 by Guo et al. [28], including 49 studies that aimed at identifying significant risk factors associated with CRBSI in hemodialysis patients demonstrated that catheter type is a significant risk factor for CRBSI (OR: 3.83, 95% CI[2.13-6.87]), but anemia did not significantly impact the risk of CRBSI (OR=1.36, 95% CI [0.15, 11.92]). In Uganda, Nanyunja et al. [13] showed that anemia at baseline was associated with a five-fold higher risk of CRBSIs during a 6-month prospective cohort of 121 patients. Similarly, in Ethiopia, Weldetensae et al. [12] found that anemia is associated with a three-fold higher risk of CRBSI in their retrospective cross-sectional study involving 353 catheter-dependent hemodialysis to determine the burden of CRBSI and related risk factors. However, anemia is a common finding in ESRD patients receiving hemodialysis. Therefore, the distribution may not be uniform among participants. In this study, for instance, all 19 cases of CRBSIs occurred in anemic patients, which could explain the observed association between anemia and CRBSIs.

This study was characterized by the predominance of gram-negative bacteria, accounting for 52.63% of cases. Similar findings were reported in other African countries, namely Ghana, Ethiopia, and Uganda, accounting for 53%, 57.6%, and 60.3% of isolates, respectively [12-13,29]. Additionally, in Italy, Mandolfo et al. [30] revisited the etiology of CRBSIs in renal units over eight years. They reported intensification of CRBSI attributed to enterobacterial and a non-statistically significant rising trend in the number of gram-negative bacteria over time from 37.8% to 44.3% coupled with a parallel reduction in the percentage of gram-positive CRBSI from 59.5% to 54.1% that is consistent with changing epidemiology of CRBSI etiologies [30]. This should raise awareness among clinicians to consider both the gram-negative and gram-positive bacteria while initiating empirical therapy for patients suspected to have CRBSI with pending culture results.

This study's limitations include its small sample size and the fact that it was conducted in a single teaching hospital with a large hemodialysis unit, which may limit the generalizability of the results to other dialysis units in the country.

## Conclusions

In conclusion, catheter-related bloodstream infections (CRBSIs) occurred at 0.78 episodes per 1,000 catheter-days. Both catheter type and anemia were strongly associated with CRBSIs, and gram-negative bacteria were the most commonly identified organisms. The study results highlighted a low baseline incidence, a high initiation of dialysis using catheters, and higher hospitalization rates. These findings underscore the need to prioritize resources toward arteriovenous fistula (AVF) creation for suitable patients and to reinforce best infection prevention and control (IPC) practices in the renal unit to mitigate morbidity, mortality, hospitalization, and costs associated with CRBSIs. Future studies could further investigate the Minimum Inhibitory Concentration (MIC) and shed light on the organism's drug resistance, as well as the healthcare burden posed by hospital-acquired infections.
